# The gut-lung axis in COPD: immunomodulatory roles of gut microbiota and novel therapeutic strategies

**DOI:** 10.3389/fimmu.2026.1733726

**Published:** 2026-01-27

**Authors:** Feng-Xian Ni, Hui-Xian Wang, Jie Hu, Pei-Sheng Chen, Pan Xu, Hui-Hui Chen, Ze-Bo Jiang, Dong-Hui Huang

**Affiliations:** 1 Zhuhai Hospital of Integrated Traditional Chinese and Western Medicine, Zhuhai, Guangdong, China; 2 Department of Respiratory Medicine, The Zhuhai Hospital of Guangdong Provincial Hospital of Chinese Medicine, Zhuhai, Guangdong, China

**Keywords:** chronic obstructive pulmonary disease, gut microbiota, gut-lung axis, immunomodulation, microbial metabolites, nanoparticles, probiotics, short-chain fatty acids

## Abstract

Chronic Obstructive Pulmonary Disease (COPD) is a progressive respiratory disorder characterized by persistent airflow limitation and systemic inflammation, with accumulating evidence implicating gut microbiota dysbiosis as a key modulator of disease pathogenesis via the gut-lung axis. This review synthesizes current knowledge on the bidirectional communication between the gut and lungs, highlighting how microbial metabolites—particularly short-chain fatty acids (SCFAs), tryptophan derivatives, and bile acids—regulate pulmonary immunity through G-protein-coupled receptors, histone deacetylase inhibition, and aryl hydrocarbon receptor signaling. Dysbiosis-driven disruptions in these pathways exacerbate neutrophilic inflammation, impair regulatory T-cell function, and sustain TLR4/NF-κB activation, amplifying lung tissue damage and remodeling. Therapeutic strategies targeting the gut-lung axis show promise in restoring microbial homeostasis and mitigating COPD progression. Probiotics (e.g., *Lactobacillus* and *Bifidobacterium*), prebiotics (e.g., inulin), and dietary interventions (e.g., high-fiber diets) enhance SCFA production, strengthen epithelial barriers, and suppress pro-inflammatory cytokines. Advanced approaches, including fecal microbiota transplantation, nanotechnology-enabled metabolite delivery (e.g., dendrimer-complexed indole-3-acetic acid), and traditional Chinese medicine (TCM) formulations (e.g., the postbiotic formulation Qipian), demonstrate efficacy in preclinical and clinical studies by synchronizing gut-lung microbiota and inhibiting inflammatory pathways. Despite these advances, challenges remain in translating findings to clinical practice, including methodological heterogeneity, antibiotic and corticosteroid confounding, and inter-individual microbiota variability. Future research must integrate multi-omics technologies, validate biomarkers (e.g., Bacteroidales/*Lactobacillus* ratio, SCFA levels), and develop personalized interventions to bridge the bench-to-bedside gap. Harnessing the gut-lung axis offers transformative potential for COPD management, shifting the paradigm from symptomatic treatment to disease-modifying strategies rooted in microbiome immunology.

## 1 Introduction

Chronic Obstructive Pulmonary Disease (COPD) represents a major global health burden, currently ranking as the third leading cause of death worldwide and affecting more than 300 million individuals (1). It is a progressive condition marked by persistent respiratory symptoms and irreversible airflow limitation, primarily due to chronic inflammation in response to inhaled irritants, most notably cigarette smoke (2). In addition to its pulmonary effects, COPD is increasingly understood as a systemic disorder associated with numerous extrapulmonary complications, including cardiovascular disease, metabolic syndrome, and musculoskeletal impairment, all of which contribute to increased morbidity and reduced quality of life (3).

Conventional pathophysiological models have emphasized local processes such as smoke-induced inflammation, oxidative stress, protease-antiprotease imbalance, and eventual tissue damage (4). However, growing evidence indicates that these mechanisms are part of a broader biological network involving systemic inflammation. Circulating inflammatory mediators originating in the lungs can affect distant organs, contributing to the multisystemic nature of COPD. Notably, this systemic inflammation often continues even after smoking cessation, implying self-sustaining mechanisms independent of ongoing exposure.

A significant paradigm shift has emerged with the growing recognition of the gut-lung axis—a bidirectional communication system between the gastrointestinal and respiratory tracts (5). This concept aligns with the ancient Traditional Chinese Medicine (TCM) doctrine of an “interior-exterior relationship between the lung and large intestine” (6). While the lung harbors its own microbiota, which is also altered in COPD, this review will focus primarily on the immunomodulatory role of the gut microbiota and the gut-lung axis, as the gut represents the largest reservoir of microbes and metabolites with systemic influence. The axis operates through several mechanisms: (1) microbial metabolites, especially short-chain fatty acids (SCFAs) derived from dietary fiber fermentation; (2) trafficking of immune cells between mucosal sites; and (3) immune-modulating molecules such as deaminotyrosine (DAT).

The human gut hosts a diverse community of approximately 30–40 trillion microorganisms, collectively termed the gut microbiota, which plays a crucial role in immune development and regulation (7). Under healthy conditions, a symbiotic relationship supports nutrient metabolism, barrier function, and immune homeostasis. However, environmental factors (including antibiotics, dietary shifts, toxins, medications, stress) and systemic inflammation, can disrupt this balance, leading to dysbiosis (8). COPD patients consistently exhibit gut dysbiosis, typified by reduced microbial diversity, decreased abundance of beneficial bacteria (e.g., *Bifidobacterium*, *Lactobacillus*, and Bacteroidetes), and increased prevalence of potentially harmful taxa (e.g., Proteobacteria and Enterobacteriaceae) (9).

These microbial alterations have profound immunological consequences. The gut microbiota modulates systemic immunity via: (1) immunoregulatory metabolites (SCFAs, tryptophan derivatives, bile acids); (2) maintenance of epithelial barrier integrity; and (3) interactions with pattern recognition receptors on immune cells. SCFAs, including acetate, propionate, and butyrate, serve as key mediators, binding to G-protein-coupled receptors (GPR41, GPR43, GPR109a) and inhibiting histone deacetylases (10). Through these pathways, SCFAs influence dendritic cells (DCs), macrophages, and T cells, promoting anti-inflammatory responses and maintaining immune balance in the lungs (11). It is noteworthy that while the luminal concentration of SCFAs in the gut is high (millimolar range), their systemic levels are considerably lower, prompting ongoing investigation into the precise mechanisms of their distal actions, which may involve the modulation of immune cell precursors in the gut.

Recent pioneering work has further elucidated the mechanisms linking gut microbiota to COPD. A 2025 investigation on “Qipian”, a postbiotic formulation based on a mixed bacterial lysate, showed that modulating gut microbiota (e.g., increasing Bacteroidetes and *Lactobacillus*) improved lung function, reduced alveolar damage, and attenuated airway inflammation in smoke-induced COPD mice (12). These effects were mediated via suppression of the Toll-like receptor 4/nuclear factor kappa B (TLR4/NF-κB) pathway and downstream pro-inflammatory cytokines (TNF-α, IL-1β, IL-17). Notably, the study reported synchronized microbial changes across organs—highlighting the interconnectedness of gut and lung microbiota.

Therapeutic targeting of the gut-lung axis offers promising avenues for COPD management. Interventions such as probiotics, prebiotics, synbiotics, dietary adjustments, and fecal microbiota transplantation (FMT) are under active investigation (13). Innovative approaches, including nanozyme-probiotic delivery systems that improve probiotic survival and targeted delivery, show significant potential (14). Additionally, combining PD-1 inhibitors with microbiota transplantation and γδ T-cell therapy—already effective in oncology—may hold promise for chronic inflammatory conditions like COPD (15). Despite these advances, challenges remain in translating research into clinical practice. The complexity of host-microbe interactions, interpersonal variability in microbiota composition, and a scarcity of large-scale human studies impede progress (16). A deeper understanding of how specific microbial taxa and metabolites affect COPD progression and exacerbations is needed. Integrating multi-omics technologies—metagenomics, metabolomics, proteomics, and transcriptomics—with systems biology will be essential to unravel these complexities and identify novel therapeutic targets (17–19).

While previous reviews have established the association between gut dysbiosis and COPD, this article delves deeper into the specific pharmacological mechanisms by which microbial metabolites exert their effects on distal pulmonary immunity. We place a special emphasis on emerging therapeutic platforms, including engineered probiotics and targeted nanocarriers, which are poised to overcome the bioavailability challenges of microbiota-based therapies. Furthermore, we provide a critical appraisal of the translational pathway, from mechanistic discovery to clinical application, incorporating the latest insights from multi-omics studies and innovative model systems. This review thereby offers a forward-looking roadmap for future pharmacology and drug development targeting the gut-lung axis in COPD.

## 2 Gut dysbiosis and immune inflammation in COPD

**Figure 1:**
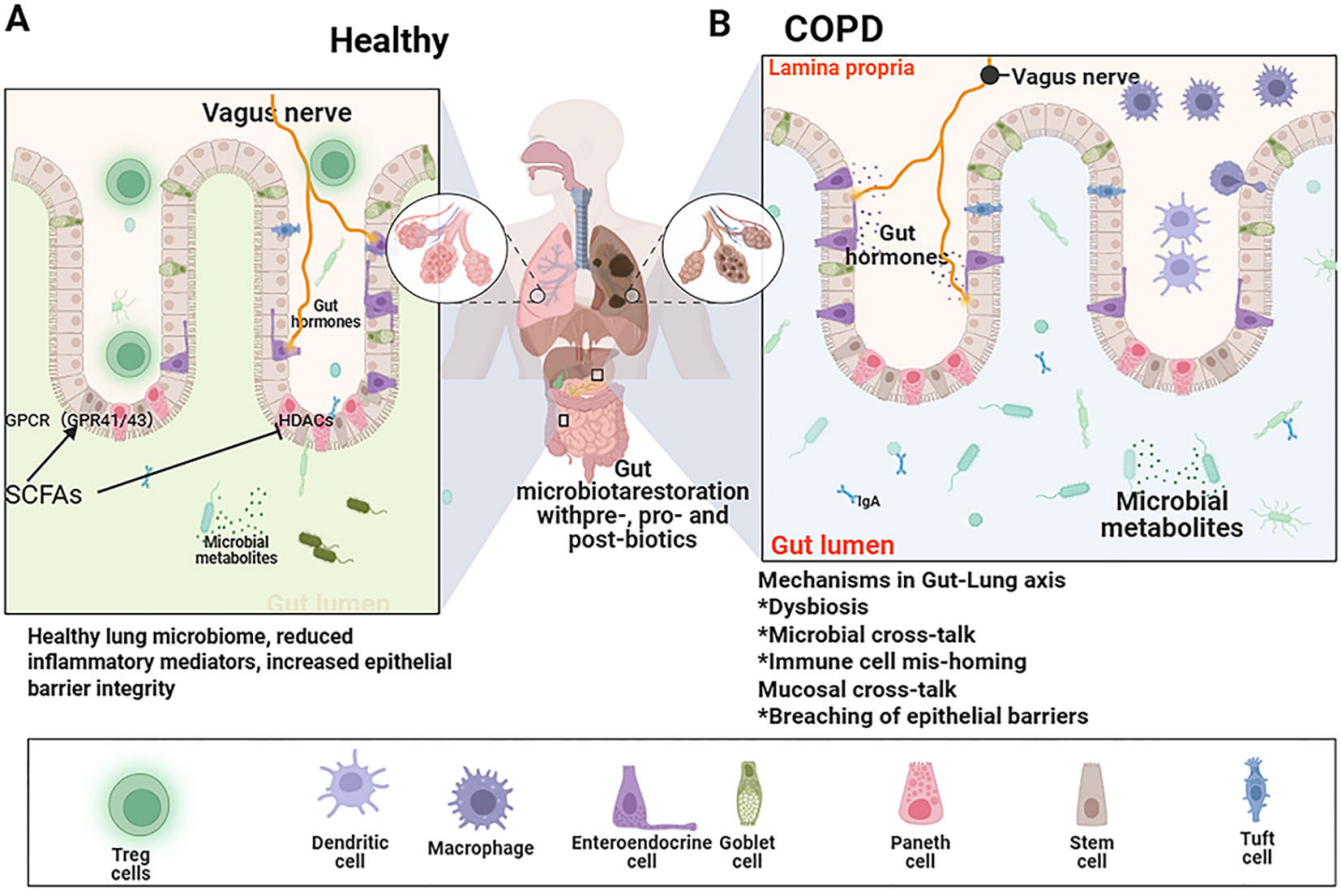
The bidirectional gut-lung axis in COPD: mechanisms and consequences. Schematic illustration contrasting a healthy state (Left) with the COPD state (Right). **(A)** In health, a diverse gut microbiota, rich in beneficial SCFA-producing taxa (e.g., *Bifidobacterium*, *Lactobacillus*, Bacteroidetes), maintains intestinal barrier integrity. Microbial metabolites, particularly SCFAs, enter circulation and promote lung immune homeostasis by inhibiting histone deacetylases (HDACs), activating G-protein-coupled receptors (GPCRs; e.g., GPR41, GPR43), and bolstering anti-inflammatory regulatory T cells (Tregs). **(B)** In COPD, gut dysbiosis is characterized by reduced diversity, a decline in beneficial bacteria, and an expansion of potential pathobionts (e.g., Proteobacteria). A compromised intestinal barrier allows translocation of microbial products like lipopolysaccharide (LPS) into the circulation. This leads to reduced systemic SCFA levels and increased pro-inflammatory signals. These factors drive pulmonary inflammation via activation of the TLR4/NF-κB pathway, promoting the recruitment and activation of neutrophils, M1 macrophages, and Th17 cells, while impairing Treg function, ultimately resulting in persistent inflammation, tissue damage, and airflow limitation.

COPD is increasingly recognized as a systemic inflammatory disorder characterized by progressive airflow limitation and irreversible lung tissue damage, with gut dysbiosis emerging as a key modulator of disease pathogenesis via the gut-lung axis (20). This bidirectional communication pathway enables intestinal microbiota to remotely influence pulmonary immune homeostasis through microbial metabolites, immune cell trafficking, and epithelial barrier regulation, thereby shaping the function of diverse immune cell populations in the lung microenvironment. This section delineates the characteristic disruptions in the gut microbial ecosystem in COPD and elucidates how these alterations drive the dysfunction of key immune cells in the lung via the gut-lung axis, establishing a self-perpetuating cycle of inflammation and impaired repair. The contrasting states of a healthy versus a COPD-affected gut-lung axis are schematically illustrated in **Figure 1**.

### 2.1 Gut dysbiosis in COPD: an overview

Patients with COPD exhibit a distinct and progressive gut dysbiosis that correlates with disease severity. This dysbiosis is characterized by three key features: (1) a loss of overall microbial diversity, (2) a depletion of beneficial commensal bacteria, and (3) an expansion of pro-inflammatory taxa. Reduced α-diversity, typically quantified by indices such as Chao1 (richness) and Shannon (richness and evenness), is closely linked to COPD severity and the frequency of acute exacerbations. Patients with severe COPD exhibit a significant reduction in gut microbial phylogenetic richness compared to healthy controls (21, 22).

The second feature is the specific depletion of SCFA-producing bacteria, which are critical for maintaining gut barrier integrity and immune homeostasis (23). Abundances of genera such as *Lactobacillus*, *Bifidobacterium*, and *Bacteroides* are consistently decreased in COPD patients (24–27). This functional deficit in SCFA production is associated with impaired mucosal immunity and heightened systemic inflammation, two hallmarks of COPD pathogenesis (28, 29). The consequent reduction in SCFA levels not only weakens the gut barrier but also systemically deprives the lung of crucial anti-inflammatory and homeostatic signals, as detailed in Section 3.

Concurrently, there is a notable overgrowth of pathogenic or pro-inflammatory microbial taxa. The relative abundance of the phylum Proteobacteria—often linked to intestinal dysbiosis and inflammation—is substantially higher in COPD patients compared to healthy controls. Additional taxa showing increased abundance include members of the genus Streptococcus and other pro-inflammatory bacteria (30). This expansion of pro-inflammatory taxa fosters a persistent pro-inflammatory microenvironment within the gut, driving sustained immune activation that may contribute to COPD progression (23). This dysbiotic landscape, characterized by a loss of beneficial signals and a gain of inflammatory ones, sets the stage for the systemic immune dysregulation observed in COPD. **Table 1** summarizes the key microbial alterations in COPD patients.

**Table 1:** Key microbial alterations in COPD patients.

Taxonomic level	Specific changes	Direction in COPD	Functional implications	Reference
Phylum	Bacteroidetes	Decreased	Reduced SCFA production	(31)
Phylum	Firmicutes	Decreased	Altered fermentation	(22)
Phylum	Proteobacteria	Increased	Enhanced inflammation	(32)
Genus	*Lactobacillus*	Decreased	Impaired immunoregulation	(32–34)
Genus	*Bifidobacterium*	Decreased	Reduced gut barrier integrity	(24, 35)
Genus	*Haemophilus*	Increased	Associated with lung function decline	(36, 37)
Family	Lachnospiraceae	Decreased	Diminished butyrate production	(35)

### 2.2 Pulmonary immune cell dysfunction and gut-mediated influences

**Figure 2:**
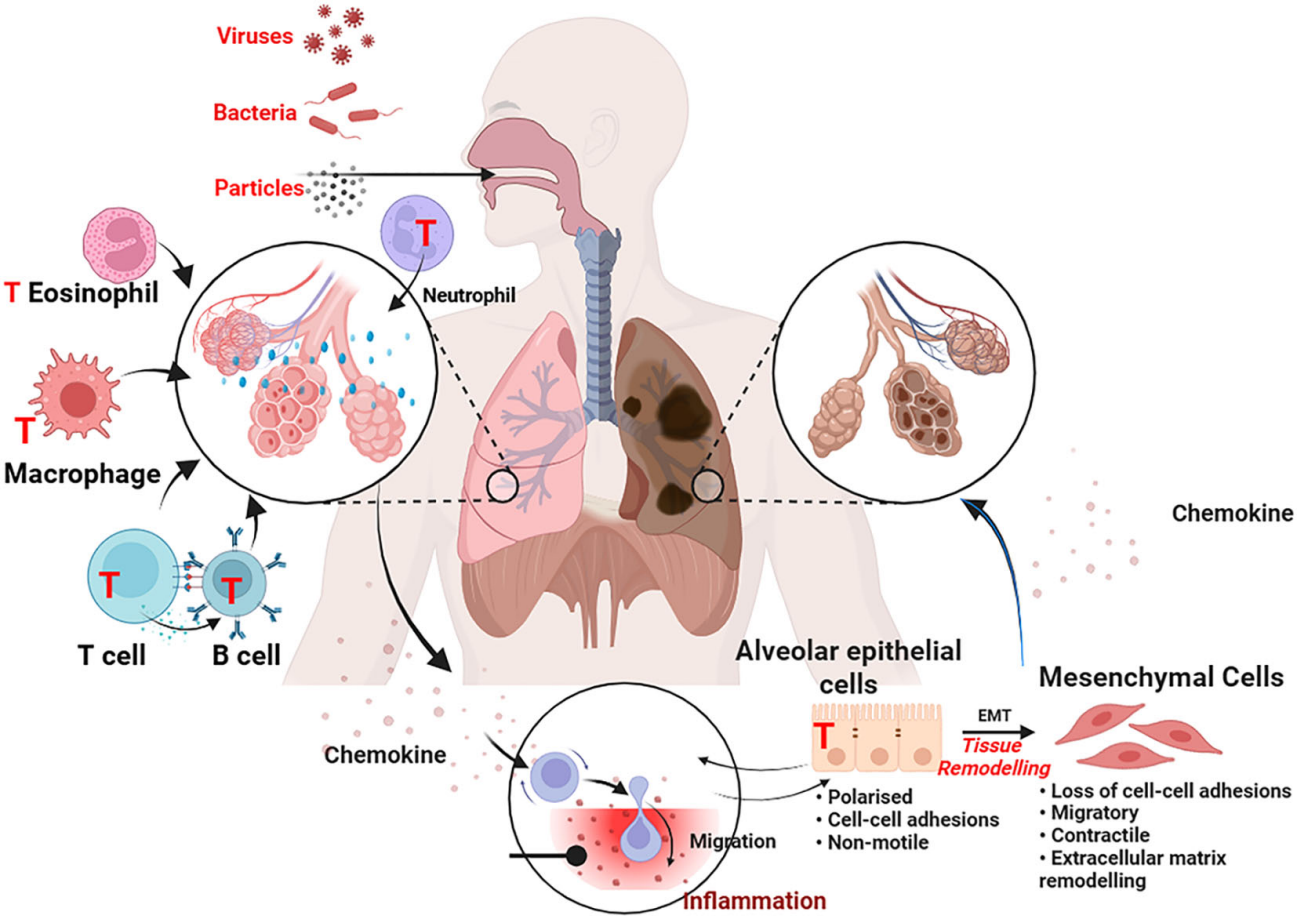
Immunoregulatory cells in COPD: roles in pathogenesis and as therapeutic targets. The pulmonary immune microenvironment in COPD is dominated by the aberrant activation and dysfunction of multiple immune cell populations. Neutrophils release ROS, NE, MMP-9, and NETs, causing tissue damage. Macrophages are skewed towards a pro-inflammatory M1 phenotype, while dysregulated M2 macrophages contribute to fibrosis. T Lymphocytes show a skewed profile with expanded cytotoxic CD8^+^ T cells, increased Th1/Th17 cells, and deficient Tregs. Dendritic Cells (DCs) and Myeloid-Derived Suppressor Cells (MDSCs) further contribute to immune dysregulation. Alveolar Epithelial Cells are key targets of damage and contributors to inflammation. Red “T” symbols indicate potential therapeutic targets for modulating each cell type. The background depicts damaged alveoli and chronic inflammation.

The pulmonary pathology of COPD is driven by the aberrant activation and impaired resolution of both innate and adaptive immune cells. We now detail the role of key cellular players, highlighting the emerging links to gut-derived signals. An overview of the major immune cells involved in COPD pathogenesis and their potential as therapeutic targets is provided in **Figure 2**.

#### 2.2.1 Neutrophils: drivers of inflammation and tissue destruction

Neutrophils are critical for host defense but become pathogenic in COPD due to sustained activation (38). In patients with COPD, neutrophil counts are significantly elevated in bronchoalveolar lavage fluid (BALF), sputum, and lung tissue, with numbers correlating with disease severity and exacerbation frequency (39). once activated, neutrophils release a repertoire of cytotoxic molecules, including reactive oxygen species (ROS), proteolytic enzymes (neutrophil elastase, matrix metalloproteinase-9 (MMP-9)), and neutrophil extracellular traps (NETs), which collectively drive tissue damage and emphysematous changes (40, 41). Notably, neutrophil dysfunction in COPD extends beyond hyperactivation: impaired phagocytosis and chemotaxis reduce their ability to clear pathogens, increasing susceptibility to infections and exacerbations (42, 43).

Gut-lung axis link: The hyperactive state of pulmonary neutrophils is systemically influenced by gut health. Circulating LPS derived from a dysbiotic gut can ‘prime’ neutrophils, lowering their activation threshold. Furthermore, the systemic deficit in gut-derived SCFAs removes a crucial brake on neutrophil chemotaxis and ROS production, thereby exacerbating their damaging potential in the lung (44–46).

#### 2.2.2 Macrophages: orchestrators of inflammation and remodeling

Alveolar macrophages (AMs) are activated by cigarette smoke and microbial products. In COPD, there is a skewing toward pro-inflammatory M1 polarization, characterized by increased expression of inducible nitric oxide synthase (iNOS), TNF-α, IL-6, and IL-1β, which amplify inflammation and oxidative stress (47).M2 macrophages, associated with tissue repair, are also dysregulated (46, 48). Excessive or prolonged M2 activation can drives aberrant tissue remodeling via transforming growth factor-beta (TGF-β) and MMP-12, leading to airway fibrosis (49). Macrophage dysfunction in COPD is also marked by impaired efferocytosis—the clearance of apoptotic cells—which leads to the accumulation of necrotic debris and persistent inflammation (50).

Gut-lung axis link: The polarization state of pulmonary macrophages is susceptible to remote reprogramming by gut metabolites. Butyrate and other SCFAs are potent inducers of an anti-inflammatory and pro-resolutive phenotype in macrophages, primarily through HDAC inhibition (10). The systemic SCFA deficiency characteristic of COPD thus cripples a key signal that would otherwise help temper M1-driven inflammation and promote tissue repair in the lung.

#### 2.2.3 T cells: mediators of adaptive immune dysregulation

Adaptive immune responses mediated by T cells are central to COPD pathogenesis, CD8^+^ T cells are particularly abundant in the lungs of patients with COPD, contributing to alveolar epithelial cell death and emphysema severity (51). CD4^+^ T cells in COPD are polarized toward pro-inflammatory Th1 and Th17 phenotypes, with reduced anti-inflammatory T regulatory (Treg) cells, creating a Th17/Treg imbalance that perpetuates inflammation (52). Treg cells, which suppress immune responses, are numerically and functionally deficient in COPD, failing to constrain Th1 and Th17 activity.

Gut-lung axis link: The systemic deficit of SCFAs, particularly butyrate, impairs the epigenetic programming of Treg differentiation in the gut and their subsequent trafficking to the lung. Concurrently, gut dysbiosis can promote the priming and expansion of Th17 cells, which then migrate to the pulmonary environment, exacerbating inflammation (53).

#### 2.2.4 Dendritic cells: initiators of adaptive immune responses

DCs are professional antigen-presenting cells that link innate and adaptive immunity by capturing and presenting antigens to naive T cells, thereby initiating T cell differentiation. In COPD, DC function is dysregulated, leading to inappropriate T cell activation and polarization (54). Airway DCs in COPD exhibit an activated phenotype, expressing increased levels of costimulatory molecules (CD40, CD80, CD86) and pro-inflammatory cytokines (IL-6, IL-12, TNF-α), which promote Th1 and Th17 differentiation (55). This activation is triggered by cigarette smoke components, microbial products (e.g., LPS), and epithelial-derived cytokines such as thymic stromal lymphopoietin (TSLP) and IL-33.

Gut-lung axis link: The constant low-grade influx of microbial products (e.g., LPS) from a leaky gut provides a persistent stimulus for DC activation. Moreover, microbial metabolites can shape the development and function of both DCs and MDSCs in the bone marrow, illustrating how the gut microbiota can systemically modulate the very origin and priming of these critical innate immune cells (45).

#### 2.2.5 Myeloid-derived suppressor cells: immune suppression and inflammation

MDSCs are a heterogeneous population of immature myeloid cells with potent immunosuppressive activity, which are increasingly recognized as players in COPD pathogenesis (56). MDSCs accumulate in the peripheral blood and BALF of COPD patients, with their numbers correlating with disease severity and exacerbation risk. These cells suppress T cell proliferation and function via arginase-1, inducible iNOS, and ROS, creating an immunosuppressive microenvironment that impairs pathogen clearance and promotes chronic inflammation (57). While MDSCs initially act to limit excessive immune responses, their sustained activation in COPD leads to immune paralysis, increasing susceptibility to bacterial and viral infections. MDSCs also contribute to inflammation by secreting pro-inflammatory cytokines such as IL-6 and TNF-α, which amplify neutrophil and macrophage activation. Moreover, MDSCs promote angiogenesis and fibrosis via vascular endothelial growth factor (VEGF) and TGF-β secretion, contributing to airway remodeling (58).

Gut-lung axis link: The expansion and activation of MDSCs in COPD can be fueled by gut dysbiosis. The translocation of microbial products like LPS into the circulation may activate TLR4/NF-κB signaling in myeloid progenitors, promoting MDSC differentiation and recruitment to the lung.

### 2.3 Mechanisms of gut-lung axis in immune dysregulation

The gut-lung axis modulates pulmonary immune responses via microbial metabolites, immune cell trafficking, and epithelial barrier integrity. In COPD, gut dysbiosis disrupts this axis, leading to dysregulation of pulmonary immunity.

#### 2.3.1 Microbial metabolites: key mediators of immune regulation

SCFAs-acetate, propionate, and butyrate-are the primary metabolites produced by fermentation of dietary fiber by gut bacteria, and their levels are significantly reduced in COPD patients (59). SCFAs exert profound effects on pulmonary immune cells via G protein-coupled receptors (GPR41, GPR43) and histone deacetylase (HDAC) inhibition. Butyrate, a potent HDAC inhibitor, promotes Treg differentiation, thereby restoring the Th17/Treg balance (60). Propionate and acetate activate GPR41 and GPR43 on macrophages, inhibiting M1 polarization and promoting M2-mediated tissue repair. Additionally, SCFAs reduce neutrophil chemotaxis and ROS production, limiting tissue damage (61).

Tryptophan metabolites, including indole-3-acetic acid (IAA) regulate pulmonary immunity (62). IAA, produced by *Lactobacillus* species, inhibits pro-inflammatory cytokine production, while kynurenines activate the aryl hydrocarbon receptor (AhR) on T cells, promoting Treg differentiation and IL-22 secretion, which enhances epithelial barrier integrity (63). In COPD, depletion of tryptophan-metabolizing bacteria leads to reduced IAA and kynurenine levels, impairing AhR signaling (64). Bile acids, modified by gut bacteria, also contribute to gut-lung axis signaling. Tauroursodeoxycholic acid (TUDCA), a secondary bile acid, inhibits NLRP3 inflammasome activation in macrophages, but can also impair neutrophil function, increasing infection risk (13, 19, 65).

#### 2.3.2 Immune cell trafficking

The gut is a major site of immune cell development, and gut-derived immune cells traffic to the lung via the systemic circulation (66, 67). In COPD, gut dysbiosis alters this trafficking: depletion of beneficial bacteria reduces Treg migration to the lung, while expansion of pathogenic species increases Th17 and CD8^+^ T cell trafficking (9).

#### 2.3.3 Epithelial barrier integrity

The intestinal epithelial barrier prevents microbial translocation and maintains immune homeostasis, and gut dysbiosis impairs this barrier by reducing tight junction proteins (e.g., occludin, claudin) and increasing permeability. In COPD, increased gut permeability leads to translocation of LPS and other microbial products into the systemic circulation, where they activate TLR4/NF-κB signaling in pulmonary immune cells, amplifying inflammation (20). Beneficial gut bacteria, such as *Bifidobacterium* and *Lactobacillus*, strengthen the intestinal barrier; their depletion in COPD exacerbates microbial translocation and pulmonary inflammation (68).

#### 2.3.4 Modulation of inflammatory signaling pathways

Gut microbiota regulates key inflammatory signaling pathways in pulmonary immune cells, including the TLR4/NF-κB and NLRP3 inflammasome pathways (45). TLR4/NF-κB signaling is activated by LPS and other microbial products, leading to the transcription of pro-inflammatory genes. In COPD, gut dysbiosis and increased intestinal permeability are thought to promote the translocation of microbial products such as LPS into circulation, which can sustain systemic and pulmonary inflammation via pathways including NF-κB activation in macrophages and neutrophils, which perpetuates cytokine production and tissue damage (69). Beneficial bacteria, such as Bacteroidetes, inhibit TLR4/NF-κB signaling by secreting polysaccharide A (PSA), which activates TLR2 and suppresses NF-κB translocation, a unique immunomodulatory mechanism. The NLRP3 inflammasome, a multiprotein complex that activates caspase-1 and IL-1β secretion, is also regulated by gut microbiota. In COPD, increased gut permeability leads to activation of the NLRP3 inflammasome in macrophages, enhancing IL-1β production and inflammation (23, 70). SCFAs and bile acids inhibit NLRP3 activation, and their reduced levels in COPD contribute to inflammasome hyperactivation. Additionally, gut bacteria such as *Akkermansia muciniphila* reduce NLRP3 activation by modulating gut barrier function, highlighting the potential of microbiota-based interventions to target inflammatory pathways (71).

## 3 Mechanistic pathways of the gut-lung axis in COPD: a pharmacological perspective

**Figure 3:**
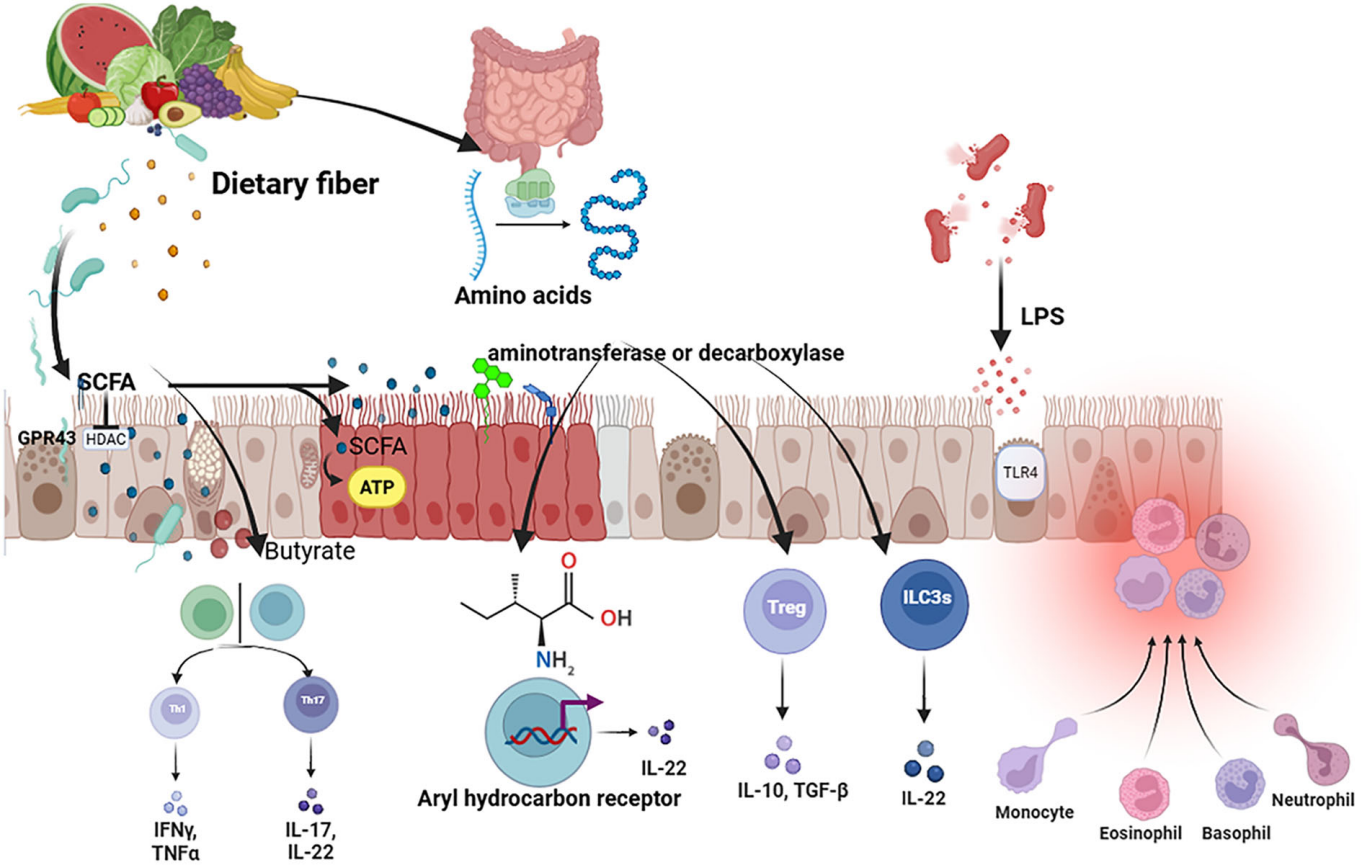
Molecular mechanisms of microbial metabolites in COPD immunomodulation. Detailed mechanistic pathways through which key gut microbial metabolites systemically influence pulmonary immunity. Short-Chain Fatty Acids (SCFAs) exert anti-inflammatory effects via two primary mechanisms: (i) Activation of G-protein-coupled receptors (GPCRs: GPR41, GPR43) on immune cells, inhibiting NF-kB signaling and proinflammatory cytokine production; (ii) HDAC inhibition (primarily by butyrate), which promotes chromatin relaxation and transcription of the Foxp3 gene, enhancing the differentiation and function of Tregs. Tryptophan Metabolites (e.g., Indole-3-acetic acid, IAA; Kynurenines) activate the Aryl Hydrocarbon Receptor (AhR) within immune cells. AhR activation promotes Treg differentiation and stimulates Group 3 Innate Lymphoid Cells (ILC3s) to produce Interleukin-22 (IL-22), which enhances epithelial barrier function and repair. In COPD, dysbiosis enables translocation of LPS, which activates the TLR4/NF-kB pathway in lung immune cells, leading to a potent pro-inflammatory response and neutrophil recruitment. The balance between these beneficial metabolite pathways and harmful LPS signaling is disrupted in COPD, favoring inflammation.

The gut-lung axis constitutes a sophisticated, bidirectional communication network. In COPD, this cross-talk is profoundly disrupted, with gut dysbiosis driving distal pulmonary pathology through well-defined molecular mechanisms. This section delineates the principal pathways through which gut-derived signals modulate lung immunity and inflammation, as illustrated in **Figure 3**.

### 3.1 Metabolite-mediated communication: molecular targets and signaling cascades

Microbiota-derived metabolites are pivotal remote controllers of pulmonary immunity, acting as potent ligands for host receptors and enzymes.

#### 3.1.1 Short-chain fatty acids: epigenetic and receptor-mediated immunomodulation

scfas, primarily acetate, propionate, and butyrate, are produced by bacterial fermentation of dietary fibers. They exert pleiotropic effects via two primary, interconnected mechanisms:

##### 3.1.1.1 G-protein coupled receptor activation

Individual SCFAs exhibit distinct receptor affinities, leading to specific immunomodulatory outcomes. Butyrate shows high affinity for GPR109a on macrophages and DCs, triggering an anti-inflammatory program that suppresses NF-κB signaling and promotes IL-10 production. Propionate, signaling predominantly through GPR41, and acetate, via GPR43, work in concert to inhibit neutrophil chemotaxis and reactive oxygen species (ROS) production. The activation of these receptors on airway epithelial cells also strengthens barrier function by upregulating tight junction proteins (71).

##### 3.1.1.2 Histone deacetylase inhibition

Butyrate is the most potent HDAC inhibitor among SCFAs. By inhibiting HDACs in CD4^+^ T-cells and antigen-presenting cells, it promotes chromatin relaxation at the Foxp3 gene locus, a master regulator for regulatory T-cell (Treg) differentiation. This epigenetic reprogramming enhances the population and suppressive function of Tregs, thereby restoring the critical Th17/Treg balance that is dysregulated in COPD. Concurrently, HDAC inhibition in AMs promotes a shift from a pro-inflammatory M1 phenotype to an anti-inflammatory, tissue-reparative M2 state (72).

A significant consideration for the HDACi mechanism is the concentration gradient; while SCFAs reach millimolar (mM) levels in the gut lumen, their systemic circulation concentrations are in the low micromolar (μM) range. T This pharmacokinetic disparity questions the feasibility of direct HDAC inhibition in distal organs like the lungs and suggests alternative mechanisms. It is hypothesized that SCFAs may exert effects through the recruitment and education of immune cells in the gut-associated lymphoid tissue (GALT), which then traffic to the lung, or that localized concentrations in specific tissue microenvironments might be higher than in general circulation.

Beyond receptor-mediated and epigenetic mechanisms, SCFAs can serve as key metabolic substrates for cellular energy production. In conditions of injury or stress, lung epithelial cells and immune cells may utilize SCFAs like butyrate—a preferred energy source for colonocytes—to fuel oxidative phosphorylation (OXPHOS). A metabolic shift away from glycolysis and towards OXPHOS is often associated with a less inflammatory cellular state, suggesting that SCFAs may also promote resolution of inflammation by modulating core cellular metabolism in the lung.

#### 3.1.2 Tryptophan metabolites and bile acids

Gut microbes metabolize dietary tryptophan into ligands for the aryl hydrocarbon receptor (AhR). AhR activation in CD4^+^ T-cells promotes their differentiation into Tregs while suppressing Th17 development, mitigating Th17/Treg imbalance (73, 74). The kynurenine pathway of tryptophan metabolism also generates AhR ligands. Bile acids, upon modification by gut bacteria, become potent signaling molecules. TUDCA, a secondary bile acid, exerts complex effects in COPD. It has been demonstrated to suppress the NLRP3 inflammasome activation in macrophages, thereby reducing the cleavage and release of pro-inflammatory IL-1β (75). However, in a paradoxical role, TUDCA can also impair AMP-activated protein kinase (AMPK) signaling in neutrophils. This disruption cripples their phagocytic capacity and oxidative burst, increasing susceptibility to secondary bacterial infections, such as those caused by *Pseudomonas aeruginosa* (76), a common pathogen in COPD exacerbations.

Deaminotyrosine (DAT), another microbiota-derived metabolite, enhances type I interferon signaling and protects against viral infections, which are major triggers of COPD exacerbations (77). **Table 2** summarizes key microbial metabolites in gut-lung communication.

**Table 2:** Key microbial metabolites in gut-lung communication.

Metabolite	Producing bacteria	Receptors/Targets	Immunological effects	Impact on lung health	Reference
Butyrate	*Faecalibacterium prausnitzii*, *Roseburia* spp.	GPR43, GPR109a, HDACs	Enhances Treg differentiation, reduces NF-κB activation	Attenuates neutrophilic inflammation, improves barrier function	(78)
Propionate	Bacteroidetes, *Akkermansia*	GPR41, GPR43	Inhibits HDAC, modulates dendritic cell function	Reduces allergic airway inflammation	(79)
Acetate	*Bifidobacterium*, *Prevotella*	GPR43	Enhances neutrophil chemotaxis, IgA production	Strengthens antiviral defense	(80)
TUDCA	*Eggerthella lenta*	AMPK	Impairs neutrophil phagocytosis	Aggravates bacterial infection (e.g., *Pseudomonas*)	(76)
DAT	*Clostridium* spp., Bacteroides spp.	Type I IFN pathway	Enhances interferon signaling	Protects against viral infections	(77)

### 3.2 Immune cell trafficking and cytokine networks

The gut microbiota systemically primes and directs the migration of immune cells. In the gut lamina propria, DCs conditioned by microbial antigens and SCFAs adopt a tolerogenic phenotype, promoting Tregs differentiation. During COPD-related dysbiosis, this process is subverted, leading to increased priming of Th17 cells, which traffic to the lungs via the CCR6-CCL20 axis and secrete IL-17, amplify neutrophilic inflammation (81). Cytokine networks further underpin this communication; SCFAs boost anti-inflammatory cytokines like IL-10, while gut dysbiosis can lead to increased levels of IL-1β and IL-17, promoting neutrophil recruitment (82).

### 3.3 Emerging mechanisms: expanding the paradigm of cross-organ communication

Recent research has uncovered several novel mechanisms that further illustrate the complexity of the gut-lung axis.

#### 3.3.1 The role of gut commensal protozoa

Moving beyond bacteria, pioneering research has identified gut-resident protozoa as key modulators of pulmonary immunity. Colonization of the gut by the rodent-specific commensal *Tritrichomonas musculis (T.mu)* triggers the activation and S1P-dependent migration of inflammatory type 2 innate lymphoid cells (iILC2s) from the gut to the lungs, where they produce type 2 cytokines, driving eosinophil accumulation (83). It is crucial to note that T. musculis is a rodent-specific commensal. While this discovery unveils a novel eukaryotic component of the gut-lung axis, the direct translation to human COPD is not yet established. This highlights the gut–lung axis as a potential conduit for eukaryotic signals, representing an emerging frontier for research rather than a confirmed mechanism in human disease.

#### 3.3.2 Bacterial extracellular vesicles

Bacteria release nanosized extracellular vehicles (EVs) that carry a cargo of microbial components (e.g., DNA, RNA, proteins). These EVs can traverse biological barriers, enter the systemic circulation, and deliver their cargo directly to cells in the lungs. Preliminary evidence suggests that bacterial EVs could serve as vectors for inter-organ signaling, potentially carrying anti-inflammatory cargo to AMs, while those from pathobionts could carry virulence factors that exacerbate inflammation (84). This represents a nascent but intriguing frontier for understanding gut-lung communication and future therapeutic design.

#### 3.3.3 Human-relevant model systems: organs-on-chips

To conclusively establish causality and dissect human-specific mechanisms, advanced model systems like organs-on-chips are invaluable. These microfluidic devices can co-culture human gut and lung organoids or epithelial cells, allowing for the real-time study of immune cell trafficking and metabolite exchange in a controlled, human-relevant system (85). While integrated gut-lung organ-chip models remain under development, they hold considerable promise for delineating human-specific causal mechanisms and serving as platforms for preclinical therapeutic screening.

The mechanistic underpinnings of the gut-lung axis in COPD highlight a complex interplay of soluble metabolites, mobile immune cells, and neural signals. The dysregulation of these pathways—SCFA-receptor signaling, AhR activation, and bile acid metabolism—offers a rich landscape of druggable targets. Future research must leverage human-relevant models, such as organ chips, to validate these mechanisms and accelerate the development of targeted therapies, including engineered probiotic consortia, metabolite-based biologics, and nanocarriers for targeted delivery to the gut or lung. A deep, mechanistic understanding of this axis is paramount for ushering in a new era of microbiome-based pharmacology for COPD.

## 4 Microbiota-targeted interventions for COPD: from microbial modulation to pharmacological translation

The delineation of the gut-lung axis has fundamentally reshaped the therapeutic landscape for COPD. Interventions aimed at restoring microbial homeostasis encompass a sophisticated arsenal that includes precision probiotics, targeted prebiotics, multi-compound traditional medicines, postbiotics, and nanotechnology-enabled delivery systems (86), as well as postbiotics—defined as inactivated microbes and/or their components—which offer a stable and safe alternative to live probiotics. This section critically evaluates these strategies, with a focused lens on their mechanistic underpinnings and potential for clinical translation.

### 4.1 Probiotics, prebiotics, and synbiotics

Probiotics (live microorganisms) and prebiotics (selective substrates) represent accessible microbiota-targeting therapies. Their synergy (synbiotics) is grounded in complementary pharmacodynamics (87).

#### 4.1.1 Probiotics: strain-specific immunomodulation

Probiotic administration, particularly with *Lactobacillus* and *Bifidobacterium* strains, demonstrates benefits in improving lung function and reducing systemic pro-inflammatory cytokines (88, 89).

*Lactobacillus* spp.: Certain strains ameliorate lung inflammation and mitigate gut dysbiosis in murine models (90). Their efficacy is linked to the enhancement of Treg responses and SCFAs production. Notably, some airway-derived *Lactobacillus* strains are potent producers of indole-3-acetic acid (IAA). Intranasal administration of these specific strains was shown to restore IAA levels, activate the IL-22 signaling pathway, and attenuate neutrophilic inflammation and emphysema in COPD models (90).

*Bifidobacterium* spp.: These probiotics primarily contribute to fortifying the intestinal barrier, reducing the translocation of pro-inflammatory microbial products like LPS, which is crucial for dampening the systemic inflammation (19). It is important to note that probiotic effects are often strain-specific, and the optimal strains, combinations, and formulations for COPD management remain an active area of investigation.

#### 4.1.2 Prebiotics: fostering a resilient microbiota

Prebiotics function as selective substrates to cultivate a beneficial gut environment. Inulin supplementation has been shown to delay symptom progression and reduce exacerbation frequency in COPD patients, primarily by stimulating SCFA-producing bacteria such as Lachnospiraceae (91). Similarly, partially hydrolyzed guar gum (PHGG), under investigation in a phase II clinical trial (NCT05126654), aims to selectively enrich *Parabacteroides goldsteinii*, a bacterium whose abundance is inversely correlated with COPD severity (92). **Table 3** lists select probiotic and prebiotic interventions in COPD.

**Table 3:** Select probiotic and prebiotic interventions in COPD.

Intervention type	Specific agent/Strain	Key findings in COPD	Proposed mechanisms	Reference
Probiotic	*Lactobacillus* spp.	Improved FEV1/FVC, reduced TNF-α, IL-6	Enhanced Treg response, increased SCFA production, pathogen exclusion	(69, 93)
Probiotic	*Bifidobacterium* spp.	Improved FEV1/FVC, reduced TNF-α, IL-6	Gut barrier fortification, immunomodulation	(94)
Prebiotic	Inulin	Slowed symptom progression and exacerbations	Stimulates SCFA-producing bacteria (e.g., Lachnospiraceae)	(95)
Prebiotic	Partially Hydrolyzed Guar Gum (PHGG)	(Under clinical trial NCT05126654)	Enrichment of *Parabacteroides goldsteinii*	(96)
Prebiotic	Resistant Starch	Reduced inflammation and emphysema in mice	Enhances microbial glucose/starch metabolism, SCFA production	(97)

### 4.2 Dietary interventions

Dietary patterns are a foundational modulator of the gut-lung axis. The Dietary Index for Gut Microbiota (DI-GM) has emerged as a validated, quantitative tool for personalizing nutritional guidance (98). A landmark 2025 analysis of NHANES data established a striking correlation: each 1-point increase in DI-GM score was associated with an 8% reduction in COPD incidence, and individuals in the highest DI-GM quartile had a 31% lower all-cause mortality (99). The mechanism is rooted in the fermentative metabolism of high-fiber dietary components, which generate SCFAs. These metabolites then systemically inhibit HDACs, activate GPCRs (GPR41, GPR43), and suppress NF-κB-driven inflammatory pathways in the lung (100). This foundational approach paves the way for personalized nutrition strategies, where an individual’s microbial profile could guide specific dietary recommendations to optimize gut and lung health.

### 4.3 Traditional Chinese medicine and compound preparations

TCM and novel composite preparations offer multi-targeted approaches to modulating the gut-lung axis (101). “Qipian,” a mixed bacterial lysate formulation classified as a postbiotic (inactivated microbes and their components), has shown efficacy in COPD models by increasing the abundance of beneficial taxa (*Bacteroidetes*, *Lactobacillus*) and inhibiting the TLR4/NF-κB signaling pathway, resulting in reduced pro-inflammatory cytokines, diminished alveolar destruction, and improved lung function (12, 102). While these preclinical findings are promising, further validation in large-scale human trials is warranted to establish clinical efficacy and optimal dosing regimens.

### 4.4 Fecal microbiota transplantation

For severe, refractory dysbiosis, FMT serves as a radical “reset” intervention. Preclinical models confirm that transplanting fecal microbiota from COPD patients into recipient mice can transfer disease phenotypes, including characteristic microbial shifts and immune dysregulation (103). While clinical trials for COPD are still nascent, this approach holds promise for fundamentally reshaping the microbial ecosystem.

### 4.5 Nanotechnology-enabled precision delivery

A significant pharmacological hurdle is the poor bioavailability and pharmacokinetic profile of many microbial metabolites. Nanotechnology offers sophisticated solutions.

#### 4.5.1 Enhanced solubility and stability

Nano-carriers like G4 PAMAM dendrimers can be complexed with hydrophobic metabolites such as indole-3-acetic acid (IAA), dramatically improving their aqueous solubility and protecting them from premature degradation (104).

#### 4.5.2 Organ targeting and prolonged retention

Formulations such as poly (lactic-co-glycolic acid) (PLGA) nanoparticles can be engineered for the sustained release of SCFAs or other agents, achieving targeted delivery to either the gut or the lung (105).

While nanotechnology offers promising solutions for bioavailability, its clinical translation necessitates a thorough evaluation of safety. Key concerns include the potential cytotoxicity of certain materials, long-term accumulation in tissues, and inadequate biodegradation. The inflammatory response to nanoparticles, especially in the already compromised COPD lung, must be carefully assessed. Future work must therefore focus on designing biocompatible, biodegradable carriers with well-understood clearance pathways, a critical prerequisite for their successful clinical translation to ensure therapeutic benefits outweigh potential risks.

## 5 Clinical translation and challenges

The delineation of the gut-lung axis discoveries into clinical practice is fraught with multifaceted challenges (106). This section critically examines the current landscape, focusing on biomarkers development, personalized medicine, and the significant biological, methodological, and regulatory hurdles.

### 5.1 Biomarker development: from taxonomic census to functional metabolomics

The identification of reliable biomarkers is the cornerstone of precision medicine for COPD.

#### 5.1.1 Microbial signature-based biomarkers

Taxonomic shifts provide the first layer of biomarker discovery. Ratios such as the Bacteroidales/*Lactobacillus* index have been correlated with COPD severity and exacerbation risk (22).

#### 5.1.2 Functional metabolomic biomarkers

Functional metabolites provide a more dynamic readout. Levels of fecal SCFAs are consistently reduced in COPD patients and inversely correlate with systemic inflammation (84).

Personalized medicine: The inherent heterogeneity of COPD demands tailored strategies. The DI-GM enables the design of individualized dietary plans (107). Probiotic and prebiotic interventions are advancing toward greater specificity, guided by baseline microbiome profiling (108). This integrated approach, combining biomarker profiling with targeted interventions, represents the future of microbiome-based personalized medicine for COPD (19). The integration of such biomarkers into clinical practice could enable a more precise stratification of COPD patients, moving beyond the current GOLD classification towards a ‘treatable traits’ approach that includes microbiota and metabolic status. **Table 4** lists promising microbiota-based biomarkers in COPD.

**Table 4:** Promising microbiota-based biomarkers in COPD.

Biomarker type	Specific marker	Association with COPD	Clinical utility	Sample source	Reference
Microbial Ratio	Bacteroidales/*Lactobacillus*	Increases with disease severity	Prognostic stratification	Feces, BALF	(109)
Microbial Taxa	Bacteroides *vulgatus*	Decreased in COPD	Diagnostic accuracy	Feces, saliva	(19)
Microbial Taxa	*Clostridium* spp.	Decreased in COPD	Diagnostic accuracy	Feces, sputum	(110)
Microbial Taxa	Lachnospiraceae	Decreased in COPD	Diagnostic accuracy	Feces, BALF	(22)
Metabolite	Short-chain fatty acids (SCFAs)	Reduced (40-50%)	Monitoring inflammation	Feces	(61)
Metabolite	Tauroursodeoxycholic acid (TUDCA)	Increased with *Eggerthella lenta*	Predict infection risk	Serum, feces	(111)
Inflammatory-Microbial	Vicinamibacterales + TNF-α	Combined increase	Predict exacerbation risk	Feces, serum	(112)

### 5.2 Current limitations, challenges and future directions

The path to clinical translation is impeded by several significant barriers:

#### 5.2.1 Establishing causality

The majority of human studies are observational. While animal models recapitulate disease phenotypes and suggest causality, species-specific differences limit direct extrapolation (113). While randomized controlled trials (RCTs) can establish a causal link between an intervention and a clinical outcome, definitive molecular causality in human studies remains challenging. Advanced epidemiological methods like Mendelian randomization can provide stronger evidence for causal inference by leveraging genetic variants (111). However, the gold standard for establishing mechanistic causality often relies on a combination of human association studies and supportive data from interventional animal models, particularly gnotobiotic mice colonized with human microbiota.

#### 5.2.2 Methodological heterogeneity

A critical lack of standardization in sample collection (feces vs. mucosal biopsies), DNA extraction, sequencing, and bioinformatic pipelines hinders reproducibility and comparability of results (114).

#### 5.2.3 Antibiotic and corticosteroid confounding

The frequent use of antibiotics for managing exacerbations profoundly disrupts gut microbial diversity (115). Furthermore, a critical and often overlooked confounder is the widespread use of inhaled and systemic corticosteroids. Corticosteroids are potent immunomodulators that can directly alter the composition and function of the gut and lung microbiota (22, 116, 117). Therefore, the microbiota profile of a COPD patient is a composite of the disease itself and its treatment. Future studies must rigorously account for corticosteroid use (118). Furthermore, the pervasive use of inhaled and systemic corticosteroids in COPD management presents a major confounder, as these agents can directly modulate both gut and lung microbiota composition and host immune responses, independent of the disease process itself.

#### 5.2.4 Inter-individual variability

The gut microbiome is shaped by a multitude of factors including diet, geography, age, genetics, and concomitant medications. This vast heterogeneity challenges the development of universal interventions (119).

### 5.3 Future directions

To bridge the bench-to-bedside gap, emerging research must focus on confronting the core translational challenges.

#### 5.3.1 Establishing causal links

Future mechanistic studies should employ advanced gnotobiotic models, such as humanized microbiota mice, where germ-free animals are colonized with a defined human microbial community. Combined with antibiotic perturbation-recolonization experiments, these models can definitively validate the causal role of specific bacterial taxa or consortia in COPD pathogenesis and treatment response (120).

#### 5.3.2 Integrating multi-omics and personalizing medicine

The integration of metagenomics, metabolomics, and proteomics is essential to unravel the complex “microbiome-metabolite-host” interactions. This multi-omics approach should be applied in longitudinal cohorts to capture dynamic changes during disease exacerbation and stability. The goal is to transition towards precision microbiome medicine, where a patient’s microbial profile guides the selection of bespoke interventions (121).

#### 5.3.3 Navigating regulatory and safety hurdles

Microbiota-based therapies present unique regulatory pathways. Live Biotherapeutic Products (LBPs), FMT, and nano-formulated metabolites fall into novel regulatory categories. Key concerns include the risk of pathogen transmission, the long-term ecological stability of transplanted or supplemented microbes, and the unknown long-term safety profile of engineered nanomaterials. Developing clear regulatory frameworks and conducting rigorous, long-term safety studies are imperative for clinical adoption (122).

## 6 Conclusion and future perspectives

The delineation of the gut-lung axis has fundamentally transformed our understanding of COPD, shifting the therapeutic paradigm from a narrow, lung-centric approach to a holistic, system-wide perspective. This review consolidates compelling evidence that gut dysbiosis characterized by a loss of diversity and beneficial microbes, coupled with an expansion of pro-inflammatory taxa, orchestrates COPD progression through a complex network of metabolite signaling, immune cell dysregulation, and neuroendocrine communication.

**Figure 4:**
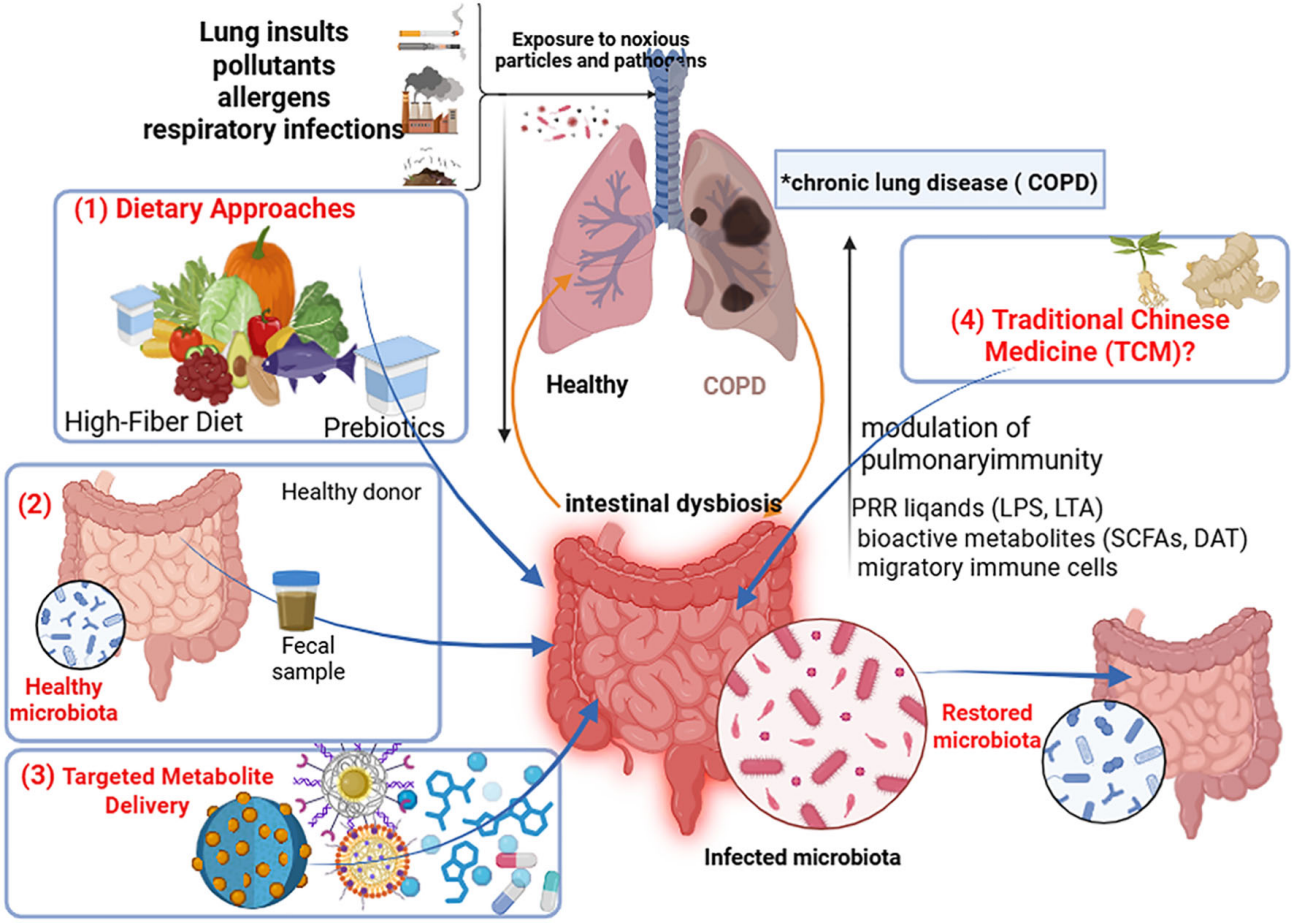
Novel microbiota-targeted therapeutic strategies for COPD via the gut-lung axis. Overview of emerging therapeutic strategies aimed at modulating the gut-lung axis for COPD management. (1) Nutritional & Microbial Interventions: Including a High-Fiber Diet to serve as substrate for commensals; Prebiotics (e.g., Inulin, GOS) to selectively promote beneficial bacteria; Probiotics (e.g., *Lactobacillus*, *Bifidobacterium* strains) to directly introduce beneficial functions. (2) Fecal Microbiota Transplantation (FMT) to reconstitute a healthy microbial community. (3) Targeted Metabolite Delivery: Utilizing Nanocarriers (e.g., PAMAM dendrimers, PLGA nanoparticles) to encapsulate and improve the delivery and bioavailability of microbial metabolites (e.g., SCFAs, IAA) to the gut or lung. (4) Traditional Chinese Medicine (TCM) & Compound Preparations: Formulations like Qipian (a mixed bacterial lysate) that modulate the microbiota and host immunity through multi-target actions. The foundation of a Personalized Medicine approach is emphasized, where microbiome profiling guides the selection of optimal combination therapies for individual patients.

Therapeutically, targeting the gut-lung axis has yielded promising strategies. Probiotics, prebiotics, and high-fiber diets aim to restore microbial equilibrium and boost the production of beneficial metabolites like SCFAs. Notably, specific TCM formulations and postbiotics, such as Qipian, have been shown to ameliorate COPD by synchronously modulating both gut and lung microbiota and inhibiting the TLR4/NF-κB signaling pathway (12). Advanced delivery systems, including nano-carriers for SCFAs and dendrimer-complexed IAA, are emerging as innovative solutions to overcome the pharmacokinetic limitations of microbial metabolites (104, 105). A summary of these novel microbiota-targeted therapeutic strategies is presented in **Figure 4**.

However, the translation of these findings into clinical practice faces significant hurdles. Key challenges include the predominance of associative human studies, methodological heterogeneity, the confounding effects of antibiotic and corticosteroids, and substantial inter-individual variability (123).

To bridge this bench-to-bedside gap, future research must focus on the following strategic areas: (1) Elucidating Causal Mechanisms with Advanced Models: Future investigations should leverage gnotobiotic models colonized with human microbiota and innovative *in vitro* platforms, such as multi-organ chips (MOoCs). These chips can simulate organ-level interactions and are crucial for moving beyond correlation and establishing causality in gut-lung communication. (2) Embracing Multi-Omics and Personalized Medicine: The integration of metagenomics, metabolomics, and proteomics is essential to unravel the complex “microbiome-metabolite-host” interactions. The goal is to transition towards precision microbiome medicine, where a patient’s microbial and metabolomic profile guides the selection of bespoke interventions. (3) Exploring Novel Therapeutic Avenues: Research should expand beyond bacterial communities to investigate the role of fungi, viruses, and gut-resident protozoa in the gut-lung axis. Additionally, the function of gut microbiota-derived extracellular vesicles (GMEVs) warrants in-depth exploration, as they represent a novel frontier for therapeutic intervention and biomarker discovery. (4) Conducting Rigorous Clinical Trials and Establishing Regulatory Frameworks: Large-scale, longitudinal randomized controlled trials (RCTs) that incorporate deep multi-omics profiling are urgently needed to validate the efficacy and safety of microbiota-targeting interventions. Concurrently, clear regulatory frameworks must be developed for live biotherapeutic products, FMT, and nano-formulated metabolites to ensure their safe and standardized clinical application.

In conclusion, the gut-lung axis has evolved from a fascinating concept into a foundation for pharmacological innovation in COPD. By shifting the therapeutic focus from managing symptoms in the lung to restoring health in the gut, we stand on the brink of a transformative era. Success will hinge on concerted, interdisciplinary collaboration to translate these insights into disease-modifying strategies that ultimately improve long-term outcomes for patients with this devastating disease.

## Glossary

AhR: Aryl Hydrocarbon Receptor
ALD: Alcoholic Liver Disease
AMPK: AMP-activated Protein Kinase
BALF: Bronchoalveolar Lavage Fluid
BCLC: Barcelona Clinic Liver Cancer
CAP: Cholinergic Anti-inflammatory Pathway
COPD: Chronic Obstructive Pulmonary Disease
CTLs: Cytotoxic T Lymphocytes
DAMPs: Damage-Associated Molecular Patterns
DAT: Deaminotyrosine
DCs: Dendritic Cells
DI-GM: Dietary Index for Gut Microbiota
EVs: Extracellular Vesicles
FEV1: Forced Expiratory Volume in 1 second
FMT: Fecal Microbiota Transplantation
FOS: Fructooligosaccharides
FVC: Forced Vital Capacity
GALT: Gut-Associated Lymphoid Tissue
GOLD: Global Initiative for Chronic Obstructive Lung Disease
GOS: Galactooligosaccharides
GPCRs: G-Protein-Coupled Receptors G
HDACs: Histone Deacetylases
HPA: Hypothalamic-Pituitary-Adrenal
IAA: Indole-3-Acetic Acid
ICIs: Immune Checkpoint Inhibitors
IDO: Indoleamine 2, 3-Dioxygenase
ILCs: Innate Lymphoid Cells
iNOS: Inducible Nitric Oxide Synthase
LPS: Lipopolysaccharide
LTB4: Leukotriene B4
MAPK: Mitogen-Activated Protein Kinase
MDSCs: Myeloid-Derived Suppressor Cells
MHC: Histocompatibility Complex
MMP: Matrix Metalloproteinase
NAFLD: Non-lcoholic Fatty Liver Disease
NASH: Non-Alcoholic Steatohepatitis
NETs: Neutrophil Extracellular Traps
NF-κB: Nuclear Factor Kappa-Light-Chain-Enhancer of Activated B Cells
NLRs: NOD-Like Receptors NOD
NLRP3: NLR Family Pyrin Domain Containing 3
NO: Nitric Oxide
PAMPs: Pathogen-Associated Molecular Patterns
PHGG: Partially Hydrolyzed Guar Gum
PSA: Polysaccharide A
PYY: Peptide YY;RCTs, Randomized Controlled Trials
ROS: Reactive Oxygen Species
SCFAs: Short-Chain Fatty Acids
TCM: Traditional Chinese Medicine
TGF-β: Transforming Growth Factor Beta
TLRs: Toll-Like Receptors
TNF-α: Tumor Necrosis Factor-Alpha
Tregs: Regulatory T Cells
TSLP: Thymic Stromal Lymphopoietin
TUDCA: Tauroursodeoxycholic Acid
VEGF: Vascular Endothelial Growth Factor.
